# Effects of *Sparassis crispa* in Medical Therapeutics: A Systematic Review and Meta-Analysis of Randomized Controlled Trials

**DOI:** 10.3390/ijms19051487

**Published:** 2018-05-16

**Authors:** Le Thi Nhu Ngoc, You-Kwan Oh, Young-Jong Lee, Young-Chul Lee

**Affiliations:** 1 Department of BioNano Technology, Gachon University, 1342 Seongnam-Daero, Sujeong-Gu, Seongnam-Si, Gyeonggi-do 13120, Korea; nhungocle92@gmail.com; 2 School of Chemical and Biomolecular Engineering, Pusan National University, 2 Busandaehak-ro, Geumjeong-Gu, Busan 46241, Korea; youkwan@pusan.ac.kr; 3 Department of Herbology, College of Korean Medicine, Gachon University, 1342 Seongnam-Daero, Sujeong-Gu, Seongnam-Si, Gyeonggi-do 13120, Korea

**Keywords:** *Sparassis crispa*, diabetes treatment, cancer therapeutic, anti-inflammatory, anti-fungal, antioxidant activity, meta-analysis

## Abstract

In this study, we investigated the therapeutic potential and medical applications of *Sparassis crispa* (*S. crispa*) by conducting a systematic review of the existing literature and performing a meta-analysis. The original efficacy treatment of the mushroom extract is considered primarily and searched in electronic databases. A total of 623 articles were assessed, 33 randomized controlled experiments were included after the manual screening, and some papers, review articles, or editorials that did not contain data were excluded. A comparative standard means difference (SMD) and a funnel plot between control and *S. crispa* groups were used as parameters to demonstrate the beneficial effects of *S. crispa* for diabetes and cancer treatment, as well as anti-inflammatory, anti-fungal and antioxidant activities. The meta-analysis was carried out using Review Manager 5.1 software. Although for therapeutic diabetes there was heterogeneity in the subgroup analysis (I^2^ = 91.9%), the overall results showed statistically significant SMDs in major symptoms that decreased serum insulin levels (SMD = 1.92, 95% CI (1.10, 2.75), I^2^ = 0%), wound rates (SMD = 3.55 (2.56, 4.54), I^2^ = 40%) and contributions to an increase in nutrient intake content (SMD = 0.32 (−0.15, 0.78), I^2^ = 0%). Simultaneously, the study confirmed the utility of *S. crispa* treatment in terms of not only anti-cancer activity (reduction of tumor activity and survival of cancer cells I^2^ = 42 and 34%, respectively) but also anti-inflammatory, anti-fungal and antioxidant activities (I^2^ = 50, 44, and 10%, respectively). Our findings suggest that *S. crispa* extracts are useful for prevention and treatment of human diseases and might be the best candidates for future medicines.

## 1. Introduction

Medical mushrooms have been approved as cures in traditional East Asian therapies [1,2]. Scientists around the world have verified the unique properties of compounds extracted from mushrooms in the prevention and treatment of cancer and other chronic diseases [3].

*Sparassis crispa* (*S. crispa*) is a species of fungus belonging to the genus Sparassis, known as Cauliflower mushroom or *Sparassis latifolia*; also called by other names such as Hanabiratake in Japanese [2,3,4]. *S. crispa* is not only an edible mushroom but also a well-known medicinal mushroom that has many medical applications [3,5] (e.g., anti-tumor and anti-carcinogenic properties; anti-inflammatory, antiviral, anti-hypertensive, anti-allergic, anti-diabetic activities, and cytokine induction [1,2,3,6,7]). Recently, this mushroom has been widely utilized in Japan and Korea [3,7,8].

*S. crispa* contains highly active biological and pharmacological ingredients (e.g., β-glucan, anti-fungal compounds (sparassol, methyl-2,4-dihydroxy-6-methylbenzoate, and methyl-dihydroxymethoxy-methylbenzoate), ergosterol peroxides, and benzoate derivatives) that are useful in the treatment of human disease [3,5,9,10,11]. In particular, β-glucan can prevent and heal common health problems such as diabetes, cancer, wound healing, as well as immune system and cytokine induction [1,6,12,13,14,15]. In addition, phenolic compounds, anti-fungal substances, and other *S. crispa* extracts may be used as anti-oxidant or anti-fungal agents [3,16,17,18,19].

Several studies have indicated, through tests on either mice or human cell lines, that *S. crispa* is a potential natural source of medicinal ingredients that can contribute to the limitations and even prevention of human disease (e.g., cancer, allergies, and especially diabetic disease) [12,13,19,20] However, in individual studies, scientists have not focused on the overall assessments of the benefits of *S. crispa* in human health as a systematic review. Thus, this study reviewed randomized and controlled trials, also conducted a systematic review and meta-analysis to evaluate the statistically significant benefits of *S. crispa* in therapeutic approaches.

## 2. Results

### 2.1. Characteristics of Included Studies

**Figure 1:**
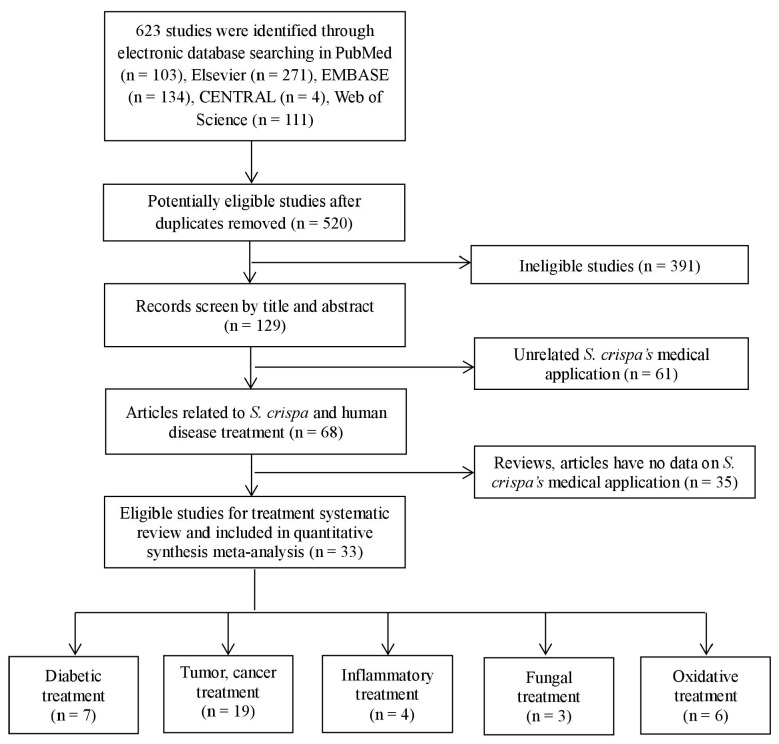
Systematic screening stages of the literature review.

Figure 1 shows the flow of candidate and eligible articles. Our searches in databases yielded a total of 623 different publications whose titles and abstracts were screened and 270 were considered relevant only by title and abstract. After reviewing these 270 full-text articles on the efficiency of *S. crispa* extracts for human-disease treatment, thirty-three articles were considered eligible and, therefore, included in the quantitative meta-analysis. One of those articles was written in Japanese [21], seven in Korean [19,22,23,24,25,26,27], and the remaining in English. Among them, some studies demonstrated more than two healing effects of the mushroom [7,26,28,29,30]. Simultaneously, the anti-diabetic, anti-tumor, anti-inflammatory, anti-fungal, and antioxidant activities of *S. crispa* were reported in seven [6,12,14,28,29,30,31], nine-teen [7,13,15,21,23,24,26,28,30,32,33,34,35,36,37,38,39,40,41], four [7,22,30,42], three [19,29,43], and six [8,25,26,27,44,45] studies, respectively.

All these thirty-three studies estimated the *S. crispa* benefits based on rats, diabetic mice, and cancer cell test (see Table 1), and provided raw data for a standardized mean difference (SMD) estimation.

### 2.2. Risk of Bias

**Figure 2:**
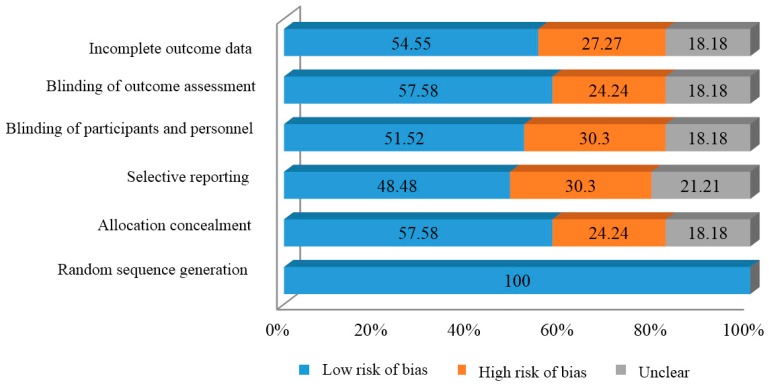
Risk of bias in individual studies graph.

To explore the validity of eligible randomized studies, the quality of bias assessment of the included studies was determined by evaluating the bias of the random sequence generation, allocation concealment, selective reporting, blinding of participants and outcome assessment, and incomplete outcome data based on three levels following the Cochrane guideline (low and high risk of bias that may indicate either lack of information or uncertainty over the potential for bias) [46]. According to Table 2 and Figure 2, almost all criteria showed a low risk of bias, especially in studies where homogeneity in the random sequence generation criteria was used. Resulting in an evident enhanced of the statistical significance of the meta-analysis.

### 2.3. Diabetes Treatment

**Figure 3:**
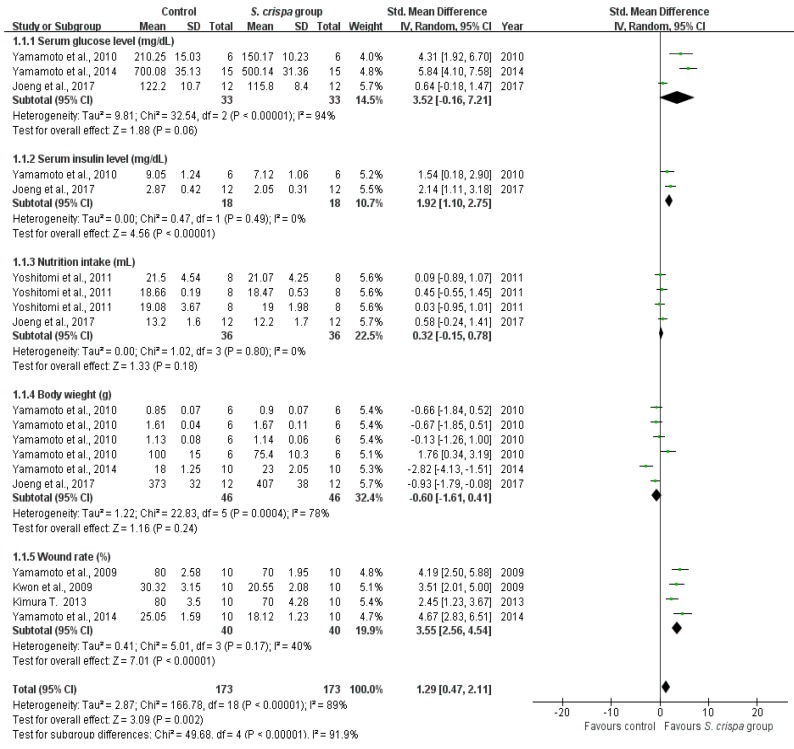
Comparison of diabetic symptoms between control and *S. crispa* groups. (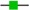): SMD of individual studies; (◆): summary SMDs of the comparison.

Anti-diabetic activity was evaluated by seven studies [6,12,14,28,29,30,31] (Figure 3 and Table 3). Most of the assays were performed in diabetic rat and mouse cells, and the treatment was assessed in five aspects: serum glucose levels (mg/dL), serum insulin levels (mg/dL), nutrition intake (mL), body weight (g) of mice or tissues before and after treatment, as well as wound healing ability (%). In addition, some individual studies have identified and demonstrated the beneficial effects of *S. crispa* in several healing aspects, so they were evaluated in various separate analyses. For instance, Yamamoto et al. showed that *S. crispa* could prevent human diabetes by reducing serum glucose levels, insulin levels, and increasing the body weight of diabetic mice [14]. In addition, Jeong et al. indicated the capability of *S. crispa* in four aspects including serum glucose and insulin levels, nutrition intake, and body weight [29].

A subgroup analysis was conducted to quantify the effect of *S. crispa* in all therapeutic approaches comparing to the control group. Anti-diabetic activity was significantly higher in the *S. crispa* group than in the control group, and results showed a significant effect of *S. crispa* in the treatment (SMD = 1.29, 95% confidence interval (CI) (0.47, 2.11), *p* < 0.00001), although a heterogeneity was observed in the subgroup analysis (heterogeneity X^2^ = 50.24, *p* < 0.00001, I^2^ = 91.9%). However, when was considered each aspect of the diabetes treatment, the comparison between serum insulin levels and wound healing rates showed significant homogeneities in all reported symptoms and presented a large SMD between the two groups ((SMD = 1.92, 95% CI (1.10, 2.75), I^2^ = 0%) and (SMD = 3.55, 95% CI (2.56, 4.54), I^2^ = 40%), respectively). Neither nutrition intakes showed heterogeneity SMD (SMD = 0.32, 95% CI (−0.15, 0.78), I^2^ = 0%). Nevertheless, serum glucose levels and body weights of rats showed high heterogeneity (I^2^ = 94% and I^2^ = 78%, respectively).

### 2.4. Cancer Treatment

**Figure 4:**
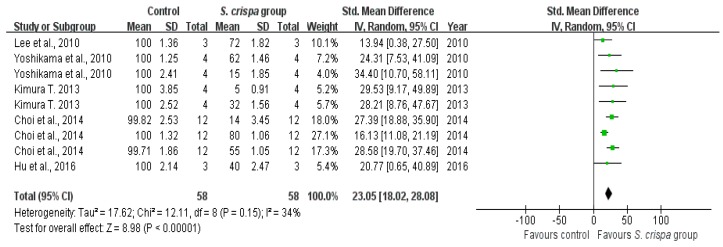
Comparison of the survival of cancer cells between control and *S. crispa* groups. (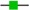): SMD of individual studies; (◆): summary SMDs of the comparison.

**Figure 5:**
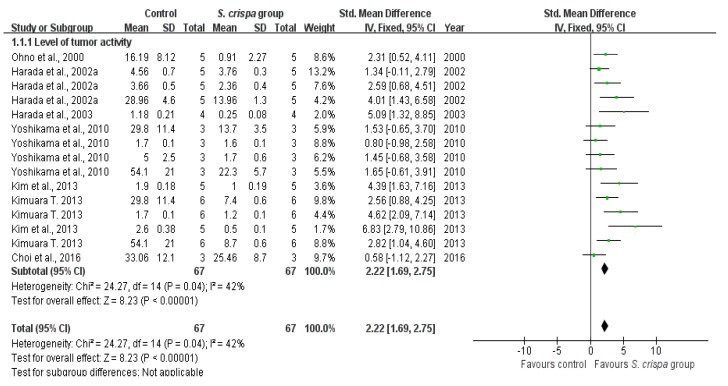
Comparison of levels of tumor activity between control and *S. crispa* groups. (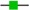): SMD of individual studies; (◆): summary SMDs of the comparison.

**Figure 6:**
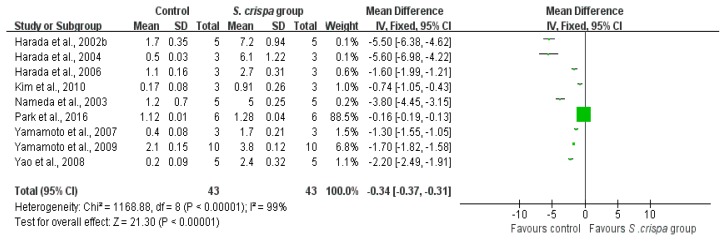
Comparison of the IFN-γ production potential between control and *S. crispa* groups. (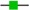): SMD of individual studies; (◆): summary SMDs of the comparison.

From nine-teen studies [7,13,15,21,23,24,26,28,30,32,33,34,35,36,37,38,39,40,41], seven reported an anti-tumor activity, five showed an inhibition of cancer cell viability, and nine indicated IFN-γ induction of *S. crispa* extracts. On the other hand, there are several reports which showed appropriate data in many respects [3,7]. In addition, when considering the capability of healing, researchers performed experiments to describe the effect of *S. crispa* extract in various types of cells [3,15,23] or to determine the therapeutic potential of different *S. crispa* extracts on a cell type [7]. Therefore, these studies have been evaluated and appeared multiple times in a comparison of this analysis. An individual analysis was applied to two groups for each of those aspects, showing significant inter-group differences (see Figure 4, Figure 5 and Figure 6).

A comparison between the control and *S. crispa* group showed a lower tumor cell activity (SMD = 2.22, 95% CI (1.69, 2.75), *p* < 0.00001); that reduction was relevant to the homogeneity in seven studie [7,13,15,24,30,34,35] (X^2^ = 24.27, I^2^ = 42%). Additionally, the heterogeneity was not significant for the survival of cancer cells (X^2^ = 12.11, I^2^ = 34%) in five studies [7,23,30,33,36], resulting in a dramatical decrease in the cancer cell viability after exposure to *S. crispa* (SMD = 21.36, 95% CI (17.91, 24.81), *p* < 0.00001). The SMD between these two groups did not show a significant of the IFN-γ induction aspect (SMD = −0.34, 95% CI (−0.37, −0.31), X^2^ = 1168.88, I^2^ = 99%).

### 2.5. Anti-Inflammatory Activity

**Figure 7:**
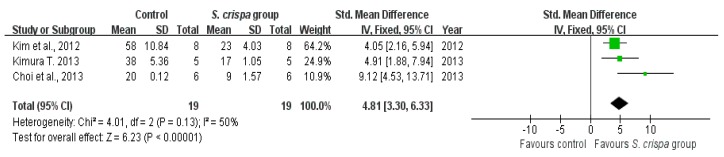
Comparison of NO production potential between control and *S. crispa* groups. (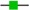): SMD of individual studies; (◆): summary SMDs of the comparison.

**Figure 8:**
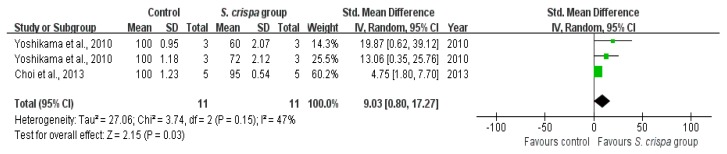
Comparison of inflammatory cells survival between control and *S. crispa* groups. (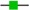): SMD of individual studies; (◆): summary SMDs of the comparison.

Data about anti-inflammatory activities of *S. crispa* extracts were reported in four studies [7,22,30,42]. According to SMDs (Figure 7 and Figure 8, and Table 3), those results demonstrated that *S. crispa* reduced inflammatory cells survival (SMD = 9.03, 95% CI (0.80, 17.27), X^2^ = 3.74, I^2^ = 47%). The heterogeneity did not exist when was compared the NO production potential between control and *S. crispa* groups, with a large effect (SMD = 4.81, 95% CI (3.30, 6.33), X^2^ = 4.01, I^2^ = 50%).

### 2.6. Anti-Fungal Activity

**Figure 9:**
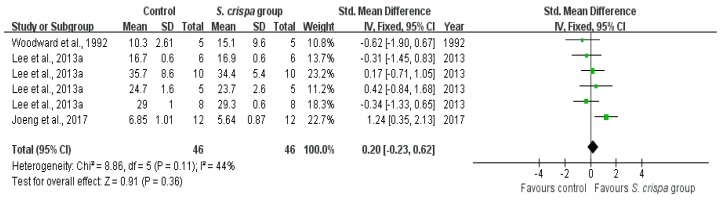
Comparison of the anti-fungal activity between control and S. crispa groups. (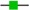): SMD of individual studies; (◆): summary SMDs of the comparison.

Anti-fungal compounds are produced by *S. crispa* in cultures and in wood decomposed naturally, as reported in three studies [19,29,43]. According to these studies, results of the meta-analysis (Figure 9) there was a favorable effect in the *S. crispa* group; the numbers of bacteria and fungi were reduced in the treatment group compared with the control group. A significant difference was found between these two groups (SMD = 0.20, 95% CI (−0.23, 0.62), X^2^ = 8.86, I^2^ = 44% < 50%).

### 2.7. Antioxidant Activity

**Figure 10:**
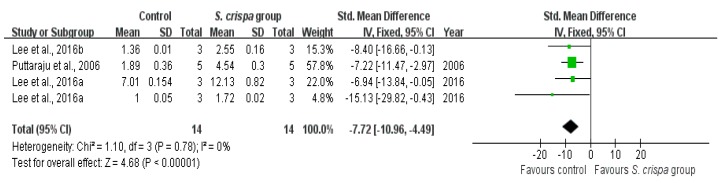
Comparison of oxidative inhibitory capacity between control and *S. crispa* groups. (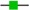): SMD of individual studies; (◆): summary SMDs of the comparison.

**Figure 11:**
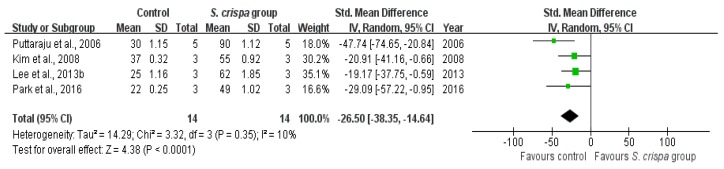
Comparison of DPPH radical scavenging activity between control and *S. crispa* groups. (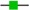): SMD of individual studies; (◆): summary SMDs of the comparison.

According to six studies [8,25,26,27,44,45], the antioxidant activity was performed through DPPH (2, 2-diphenyl-1-picrylhydrazyl radical scavenging activity and the oxidative-inhibitory capacity of phenolic compounds derived from *S. crispa*. In both cases, the *S. crispa* group reported a higher level of oxidative protection than the control group, with evidence of improving antioxidant activity (Figure 10 and Figure 11). The inhibitory activity indicated a significant homogeneity with I^2^ = 0% and X^2^ = 1.10 (SMD = −7.72, 95% CI (−10.96, −4.49), *p* < 0.00001), while the DPPH activity showed a very high statistical significance in the inter-group comparison of four relevant studies (SMD = −26.50, 95% CI (−38.35, −14.64), *p* < 0.00001, X^2^ = 3.32, I^2^ = 10%).

### 2.8. Sensitivity Analysis

**Figure 12:**
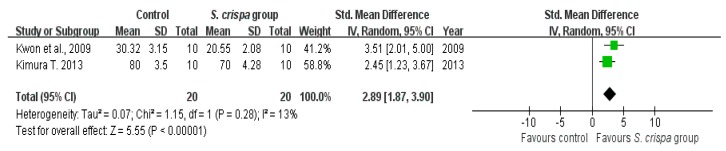
Sensitivity analysis of wound rates in diabetes treatment between control and *S. crispa* groups. (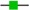): S MD of individual studies; (◆): summary SMDs of the comparison.

**Figure 13:**
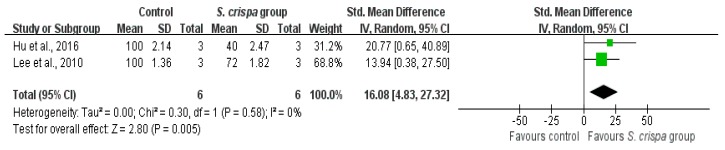
Sensitivity analysis of survival of cancer cell in cancer treatment between control and *S. crispa* groups. (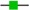): SMD of individual studies; (◆): summary SMDs of the comparison.

Studies that used the same *S. crispa* extract, similar dosages and the same experimental objects were included in the sensitivity analysis. Wound healing rates in diabetes treatment and survival of cancer cells after anti-tumor activity were analyzed (Figure 12 and Figure 13). All cases showed a high homogeneity and a high reduction of the percentages of cancer cells (SMD = 16.08 (1.83, 27.32), I^2^ = 0%), as well as an improved wound healing ability of the objects (SMD = 2.89 (1.87, 3.90), I^2^ = 13%) after treatment with *S. crispa*.

### 2.9. Bias Analysis

**Figure 14:**
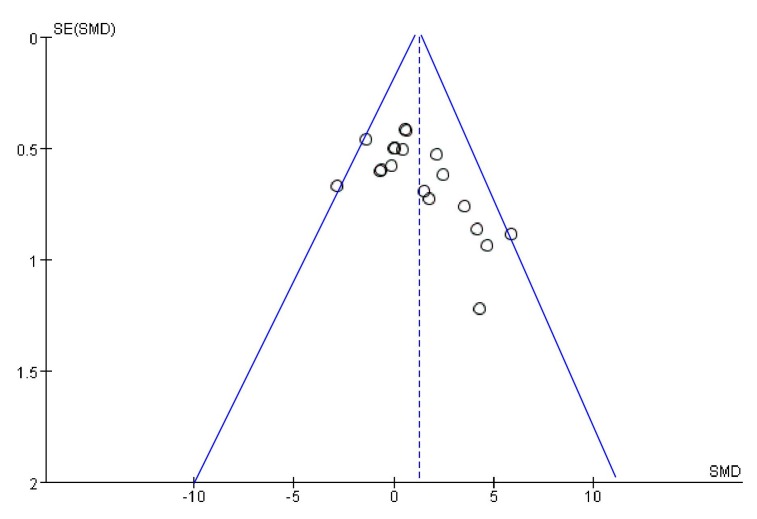
Funnel plot of studies evaluating diabetes treatment of *S. crispa*.

**Figure 15:**
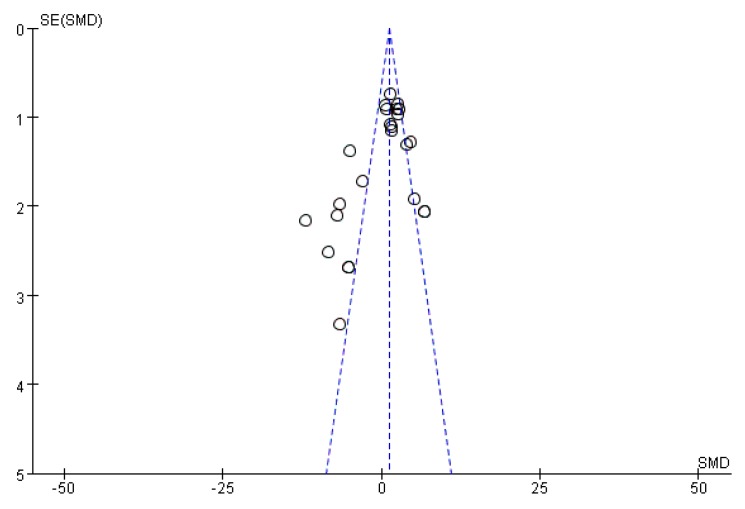
Funnel plot of studies assessment cancer treatment of *S. crispa.*

**Figure 16:**
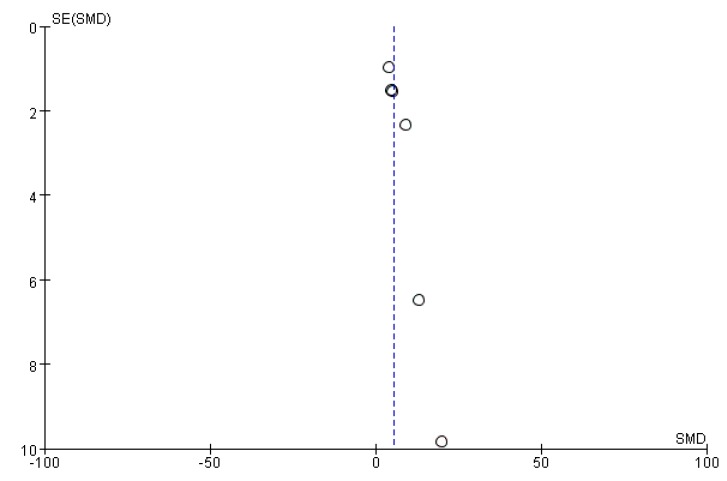
Funnel plot of studies assessment anti-inflammatory activity of *S. crispa.*

**Figure 17:**
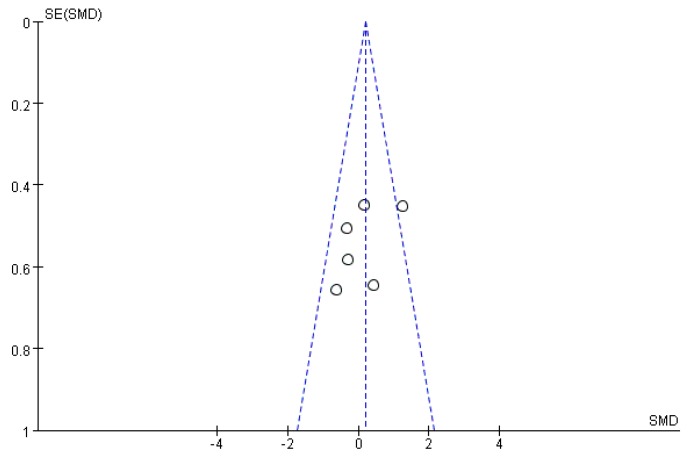
Funnel plot of studies evaluating the anti-fungal activity of *S. crispa.*

**Figure 18:**
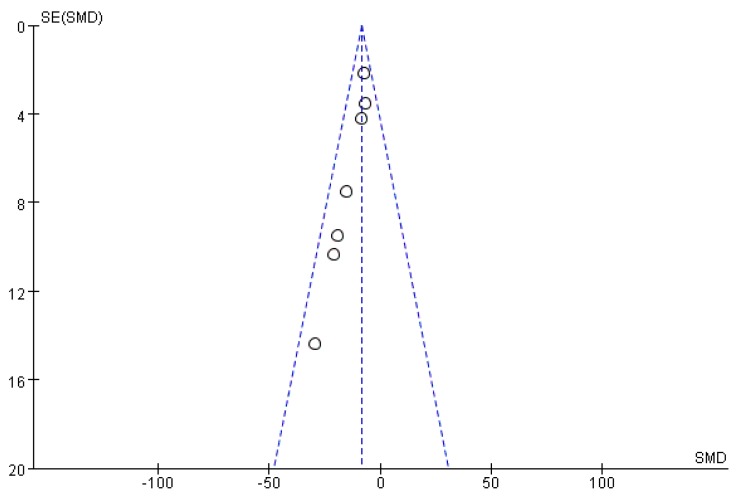
Funnel plot of studies evaluating antioxidant activity of *S. crispa.*

Funnel plots were drawn to assess the publication bias of studies on the medical applications of *S. crispa*. Figure 14 and Figure 15 are approximately symmetrical but small studies showing diabetes and cancer treatment effects of *S. crispa* remain unpublished. In contrast, Figure 16 estimated that the most important studies on anti-inflammatory activity of *S. crispa* have been missing, so the outcomes of the anti-inflammatory treatment were not highly significant statistically. Even though the funnel plots in Figure 17 and Figure 18 also demonstrated that many relevant studies have not been published yet, all the published data were statistically significant for the anti-fungal and antioxidant activity of *S. crispa* extracts.

## 3. Discussion

In this systematic review and meta-analysis of thirty-three studies on the medical application of *S. crispa* extracts were confirmed that *S. crispa* is not only an edible mushroom but also a medicinal mushroom that has been increasingly cultivated because of its potential value in traditional medicine. Indeed, *S. crispa* contains highly physiological active substances (e.g., β-glucan, phenolic compounds, chloroform extract, and some antibiotic compounds) that can support a healthy level of blood-sugar and recovery of the normal cellular immunization [3]. The beneficial effects have been demonstrated by anti-diabetic, anti-tumor, anti-inflammatory, anti-fungal, and anti-oxidant activities [1,3,11,20,47]; almost all of the typical therapeutic effects of *S. crispa* showed significant differences, relative to the control groups.

As an immunomodulating substance, β-glucan plays an important role in most healing modalities [48]. Mainly, involving an enhance of the immune response against cancer and stimulating the cells of the innate immune system [48,49,50,51]. The discovery of specific receptors for glucans in cells, as well as interactions with other receptors mainly expressed by innate immune cells, have been reported as the primary mechanism of β-glucan for regulation of anti-tumor therapy and some associated medical treatments [51]. Our meta-analysis indicated that *S. crispa* extracts had a large influence in reducing significantly cancer cells viability and tumor cells suppression (Figure 4 and Figure 5). Although, here there was not a high homogeneity in the IFN-γ production aspect (Figure 6). Moreover, the estimate as clinical evidence for a relationship between structure and activity, suggested the contributions of multiple receptor-ligand interactions in glycan-mediated immunopotentiation.

Diabetes that has been caused by a single high dose of streptozotocin is typically accompanied by symptoms such as weight loss, polyuria, hyperglycemia, and neuroendocrine dysfunction [6,14]. On the other hand, whereas wound healing progresses at an optimal rate in healthy individuals, patients with diabetes usually exhibit delayed or impaired wound healing, which is a serious high-blood-glucose-related clinical problem [52]. The present subgroup analysis did not report any significant difference (I^2^ = 91.9%) between *S. crispa* and control groups (Figure 3 and Table 3), suggesting that mushroom extracts have not had complete effects on all the diabetes symptoms. However, an individual analysis for each aspect, two topical therapy symptoms (incidence of wounds and serum insulin levels in the blood) were eliminated after treatment with *S. crispa*. In summary, *S. crispa* showed a slightly beneficial influence on diabetes therapeutics. Meanwhile, we expect that additional studies on *S. crispa* treatments will improve the accuracy of the analysis.

Additionally, the mushroom has shown an anti-inflammatory activity [1,2,47]. The present analysis estimates (Figure 7 and Figure 8, and Table 3) that studies on anti-inflammatory therapy were statistically significant, achieving a small homogeneity in the analysis. Furthermore, results also demonstrated that *S. crispa* extracts played an inhibitory role in inflammatory responses via regulation of NO production; suggesting a potential role as a component of inflammatory drugs.

Recently, some evidence has suggested that the biological actions of phenolic compounds are associated with their antioxidant capacity based on their ability to chelate metals and lipoxygenase inhibitors [19,26]. The present survey also considered and evaluated antioxidant activities of the medical mushroom by meta-analysis (Figure 10 and Figure 11). According to the coefficient of heterogeneities of the analysis (I^2^ = 0% and 10%), it was confirmed that phenolic compounds and other *S. crispa* extracts could significantly contribute to antioxidant properties; explaining the relationship between phenolic compounds and antioxidant activities, as well as anti-fungal ability.

Finally, anti-bacterial and anti-fungal compounds have been identified in *S. crispa* [43], though their utilities were reported in only three studies [19,29,43]. The analysis showed that after exposure to *S. crispa* extracts the numbers of bacteria and fungi were reduced, indicating that the mushroom, as a component of pharmaceuticals, can protect humans from bacterial and fungal contamination.

## 4. Materials and Methods

### 4.1. Methods

The study followed the Cochrane Collaboration method [46], as well as the Preferred Reporting Items for Systematic Reviews and Meta-Analyses (PRISMA) guidelines [53] to report a systematic review and meta-analysis. Also, this work was based on the protocols and reviews on medical applications of *S. crispa*. It included all the researches that assessed the ability of *S. crispa* extracts on human health treatment (i.e., diabetes and cancer treatment, anti-fungal, antioxidant, and anti-inflammatory activity). It excluded ineligible studies such as studies of the effects of mushroom in other application fields and contained inappropriate data for the analysis. It compared and analyzed the statistical significance of individual studies of the same effect of *S. crispa* for the specific therapeutics using meta-analysis. Outcomes provided an overview and a systematic assessment about the clinical efficacies of *S. crispa* based on comparing SMDs between control and *S. crispa* groups, as well as the heterogeneity coefficient (I^2^) of each analysis [54].

### 4.2. Literature Search and Data Extraction

#### 4.2.1. Database Research Strategy

The searched literature on medical applications of *S. crispa* extracts was performed in the databases PubMed (National Library of Medicine, Bethesda, MD, USA), EMBASE (Excerpta Medica database, Amsterdam, Netherlands), Elsevier (Information and Analytics, Amsterdam, Netherlands), CENTRAL (Cochrane Central Register of Controlled Trials, New York, NY, USA), and Web of Science (Institute of Scientific Information and Clarivate Analytics, Philadelphia, PA, USA), considering articles published between 1990 and 2018. Also, we made a hand searching for important conference papers, as well as checking reference lists. Combinations of the following keywords were used: *S. crispa*, *Sparassis Latifolia*, *Cauliflower mushroom*, *Hanaratake*, medical applications, immune stimulating activity, anti-tumor, anti-cancer, anti-microbial, anti-melanin, anti-metastatic, anti-inflammatory, anti-fungal, antioxidant, anti-viral, anti-diabetic, and anti-hypertensive activity. The respective reference lists of the identified papers also were searched. All articles were written in English, Korean or Japanese.

#### 4.2.2. Data Extraction

The bibliographic reference list was screened and manually selected from eligible studies from the electronic database for the meta-analysis, according to the criteria of associations between medical applications of *S. crispa* and human therapeutics. The following information from each article was obtained: article title, the name of first author, location, study year, publication year, study design, number of participants, dosage and administration, type of treatment and outcomes. These items were selected based on the presence and short descriptions of important study characteristics (e.g., title, abstract, study design, experimental object, and kind of medical application). The criteria included in the quantitative meta-analysis were empirical data that could be used to calculate the SMDs of treatments.

#### 4.2.3. Exclusion Criteria

Articles were excluded based on the following criteria: no data presentation (e.g., review articles and editorials), incomplete data, repeated and similar studies.

### 4.3. Meta-Analysis

For each analysis, we determined the effect size (SMD) and 95% CI for the comparison between control and *S. crispa* groups. The SMD was obtained by dividing the mean difference between the two groups by the pooled variance, with adjustment for small samples. We considered SMDs about 0.2 or less as small values, 0.5 as moderate values, and 0.8 or greater values as large [54]. We quantified the extension to which the observed variability between studies was due to true differences between studies using the I^2^ statistic. Heterogeneity was considered to be small when I^2^ was less than 25%, moderate when 25–50%, and large when greater than 50% [54]. The subgroup analysis assessed the overall effects in the subgroups according to the model type, kind(s) of treatment(s), and symptoms. The value of *p* < 0.05 was considered statistically significant, and bias was examined using a funnel plot.

All these analyses were performed using Review Manager [46] (version 5.3, Copenhagen: The Nordic Cochrane Centre, The Cochrane Collaboration, 2014).

## 5. Limitation of Study

The limitation of this study is the number of individual studies, which are not as large as our expectation in some respect (i.e., anti-inflammatory, anti-fungal, and antioxidant activity) leading to estimations not highly significant. Especially in the sensitivity assessment, the results might be not high accuracy because of a very small quantity of those that have the same test conditions for consideration of the sensitivity.

## 6. Conclusions

Briefly, this investigation determined that *S. crispa* is useful in medical therapeutics, each extract showed their properties and specific applications. Particularly, a meta-analysis revealed that β-glucan, which is known as the primary ingredient of *S. crispa* extract, plays an important part in the treatment of cancer and diabetes. Additionally, the analysis confirmed that β-glucan and other constituents (i.e., phthalide compounds, low-molecular-weight ingredients, and anti-bacterial substances) are used in anti-inflammatory activities, as well as antioxidant and anti-fungal immunotherapies. However, recent studies have focused on the clinical application of *S. crispa* [3,12,13,26,31,35,44]. To support our analysis, further studies to improve the statistical significance is necessary.

## Figures and Tables

**Table 1:** Summary of the characteristics of the studies included in this work.

Reference	Characteristic of Object	*S. crispa* Extract Compound	Medical Therapeutic	*n*	Dosage	Location
[6] Kwon et al., 2009	Mice	β-glucan	Anti-diabetic	10	100 µg/mL β-glucan	Korea
[32] Kim et al., 2010	Dendritic cell	β-glucan	Anti-tumor	3	100 µg/mL β-glucan	Korea
[31] Yoshitomi et al., 2011	Mice	β-glucan	Anti-diabetic	8	100 µg/mL β-glucan	Japan
[33] Lee et al., 2010	RAW 264.7 cell	β-glucan	Anti-tumor	3	250 µg/mL β-glucan	Korea
[34] Choi et al., 2016	Human fibrinogen	Wulfase	Anti-tumor	3	200 µg/mL Wulfase	Korea
[15] Harada et al., 2002a	CD 41 and CD 81 cell	β-glucan	Anti-tumor	3	200–250 µg/mL β-glucan	Japan
[35] Harada et al., 2003	Mice	β-glucan	Anti-tumor	4	25 µg/mL β-glucan	Japan
[28] Yamamoto et al., 2009	C57BL/6J cell/Mice cell line	β-glucan	Anti-diabetic Anti-tumor	10	160 µg/mL β-glucan	Japan
[7] Yoshikama et al., 2010	RAW 264.7 cell	Phthalide compounds	Anti-tumor	3–4	100 µg/mL phthalide compound	Japan
[21] Yamamoto et al., 2007	Sarcoma180 cell	*S. crispa* extract	Anti-tumor	3	35 µg/mL β-glucan	Japan
[14] Yamamoto et al., 2010	Mice	*S. crispa* extract	Anti-diabetic	6–8	100 µg/mL β-glucan	Japan
[29] Jeong et al., 2017	Mice	β-glucan	Anti-diabetic Anti-fungal	12	100 µg/mL β-glucan	Korea
[22] Choi et al., 2013	RAW 264.7 cell	β-glucan	Anti-inflammatory	3	200 µg/mL β-glucan	Korea
[23] Choi et al., 2014	A529 cell HepG2 cell AGS cell	β-glucan	Anti-tumor	12	250 µg/mL β-glucan	Korea
[30] Kimura T. 2013	Sarcoma 180 cell Mice Colon cancer cell F3444N/Rat	β-glucan	Anti-diabetic Anti-tumor Anti-inflammatory	3–5	100 µg/mL β-glucan	Japan
[42] Kim et al., 2012	Mast cell (HMC-1)	*S. crispa* extract	Anti-inflammatory	3	200 µg/mL *S. crispa* extract	Korea
[36] Hu et al., 2016	PC12 cell	β-glucan	Anti-tumor	6	250 µg/mL β-glucan	China
[44] Puttaraju et al., 2006	Mice	*S. crispa* extract	Antioxidant	3	30 µg/mL β-glucan	India
[8] Kim et al., 2008	Mice or cell	*S. crispa* extract	Antioxidant	3	100 µg/mL *S. crispa* extract	Korea
[43] Woodward et al., 1992	Botrytis cinerea	Antibiotic compounds	Anti-fungal	10	100 µg/mL antibiotic compound	United Kingdom
[13] Ohno et al., 2000	Mice	*S. crispa* extract	Anti-tumor	10	250 µg/mL *S. crispa* extract	Japan
[12] Yamamoto et al., 2014	Mice	β-glucan	Anti-diabetic	10–18	250 µg/mL β-glucan	Japan
[19] Lee et al., 2013a	Soybean	*S. crispa* extract	Anti-fungal	3	125 µg/mL *S. crispa* extract	Korea
[24] Kim et al., 2013	Raw 264.7 cell	β-glucan	Anti-tumor	5	100 µg/mL β-glucan	Korea
[37] Harada et al., 2002b	Mice	β-glucan	Anti-tumor	5	100 µg/mL β-glucan	Japan
[38] Harada et al., 2004	Mice	β-glucan	Anti-tumor	3	100 µg/mL β-glucan	Japan
[39] Harada et al., 2006	Mice	β-glucan	Anti-tumor	3	100 µg/mL β-glucan	Japan
[40] Nameda et al., 2003	Mice	β-glucan	Anti-tumor	3	50 µg/mL β-glucan	Japan
[41] Yao et al., 2008	Mice	β-glucan	Anti-tumor	10	120 µg/mL β-glucan	China
[25] Lee et al., 2016a	Soybean	β-glucan	Antioxidant	3	200µg/mL β-glucan	Korea
[26] Park et al., 2016	Mice	*S. crispa* extract	Antioxidant	6	200 µg/mL *S. crispa* extract	Korea
[27] Lee et al., 2016b	Cell	*S. crispa* extract	Antioxidant	3	50 µg/mL β-glucan	Korea
[45] Lee et al., 2013b	Mice	Phenolic compounds	Antioxidant	3	200 µg/mL phenolic compounds	Korea

**Table 2:** Risk of bias rating of each study.

Study	Random Sequence Generation	Allocation Concealment	Selective Reporting	Blinding of Participants	Blinding of Outcome Assessment	Incomplete Outcome Data
[6] Kwon et al., 2009						
[32] Kim et al., 2010						
[31] Yoshitomi et al., 2011						
[33] Lee et al., 2010						
[34] Choi et al., 2016						
[15] Harada et al., 2002a						
[35] Harada et al., 2003						
[28] Yamamoto et al., 2009						
[7] Yoshikama et al., 2010						
[21] Yamamoto et al., 2007						
[14] Yamamoto et al., 2010						
[29] Jeong et al., 2017						
[22] Choi et al., 2013						
[23] Choi et al., 2014						
[30] Kimura T. 2013						
[42] Kim et al., 2012						
[36] Hu et al., 2016						
[44] Puttaraju et al., 2006						
[8] Kim et al., 2008						
[43] Woodward et al., 1992						
[13] Ohno et al., 2000						
[12] Yamamoto et al., 2014						
[19] Lee et al., 2013a						
[24] Kim et al., 2013						
[37] Harada et al., 2002b						
[38] Harada et al., 2004						
[39] Harada et al., 2006						
[40] Nameda et al., 2003						
[41] Yao et al., 2008						
[25] Lee et al., 2016a						
[26] Park et al., 2016						
[27] Lee et al., 2016b						
[45] Lee et al., 2013b						
Risk of bias rating		Low risk of bias		High risk of bias		Unclear

**Table 3:** Summary of standardized mean difference (SMD) comparison between control and *S. crispa* groups.

Medical Application	No. of Studies	Healing Effect	*n*	SMD (95% CI)	I^2^	Total Effect
Diabetes treatment	7	Serum glucose level (mg/dL)	33	3.52 (−0.16, 7.21)	94%	SMD = 1.29 95% CI (0.47, 2.11) I^2^ = 91.9%
		Serum insulin level (mg/dL)	18	1.92 (1.10, 2.75)	0%	
		Nutrition intake (mL)	36	0.32 (−0.15, 0.78)	0%	
		Body weight (g)	46	−0.60 (−1.61, 0.41)	78%	
		Wound closure rate (%)	40	3.55 (2.56, 4.54)	40%	
Cancer treatment	19	Tumor activity	67	2.22 (1.69, 2.75)	42%	
		Cancer cell survival (%)	58	23.05 (18.02, 28.08)	34%	
		IFN- γ production (ng/mL)	43	−0.34 (−0.37, −0.31)	99%	
Anti-inflammatory activity	4	NO production (mg)	19	4.81 (3.30, 6.33)	50%	
		Inflammatory cell survival (%)	11	9.03 (0.80, 17.27)	47%	
Anti-fungal activity	3	Anti-fungal activity	46	0.20 (−0.23, 0.62)	44%	
Antioxidant activity	6	Anti-oxidant activity	14	−7.72 (−10.96, −4.49)	0%	
		DPPH (%)	14	−26.50 (−38.35, −14.64)	10%	

## Abbreviations

*S. crispa*	*Sparassis crispa*
SMD	Standardized mean difference
